# Factors underlying a disproportionate increase in hospital admissions for adrenal insufficiency in women aged 20-29 years

**DOI:** 10.3389/fendo.2023.1252577

**Published:** 2023-11-03

**Authors:** Rosemary Louise Rushworth, Henrik Falhammar, David J. Torpy

**Affiliations:** ^1^ School of Medicine Sydney, The University of Notre Dame, Australia, Darlinghurst, NSW, Australia; ^2^ Department of Molecular Medicine and Surgery, Karolinska Intitutet, Stockholm, Sweden; ^3^ Department of Endocrinology, Karolinska University Hospital, Stockholm, Sweden; ^4^ Endocrine and Metabolic Unit, Royal Adelaide Hospital, Adelaide, SA, Australia; ^5^ Discipline of Medicine, The University of Adelaide, Adelaide, SA, Australia

**Keywords:** adrenal insufficiency, adrenal crisis, psychosocial, risk factor, emerging adult

## Abstract

Since the year 2000, admissions for adrenal insufficiency (AI) and adrenal crises (AC) have shown a particular increase in young adult females. We examined data on acute non-surgical hospitalisations for AI/AC from New South Wales, Australia, to determine relevant factors that may have contributed to this increase. Data were analysed to ascertain associations between various comorbid psychosocial issues, identified by relevant ICD-10-AM codes in each record, and ACs. From 2005 to 2021. There were 877 admissions for an acute non-surgical illness in this age group. The average admission rate for females [63.5/million/year] was almost twice that for males [34.0/million/year] (p<0.01), as was the average female AC admission rate [14.7/million/year] relative to that in males [6.75/million/year] (p=NS). Infection was present in 41.6% (n=365) of the admissions and Type 1 diabetes mellitus was present in 12.2% (n=107). Overall, psychosocial factors were considered by the senior clinician to have contributed to the illness episode in 22.1% of all admissions and 29.0% of AC admissions. Having one or more psychosocial problems was associated with an AC in females (37.4%, n=49, in those having an AC, p<0.001) but not males. Females with an AC also had a higher mean composite psychosocial, psychiatric and drug/alcohol score [0.47 (0.67)] than females without an AC [0.32 (0.62) p<0.05]. No comparable associations were found in male patients. An increase in the rates of hospitalisations that included a code for at least one psychosocial problem was highly correlated with increases in admission rates for both ACs (r=0.82, p<0.001) and all AI (r=0.98, p<0.001) in females but there was no such relationship in males. This new evidence suggests that psychosocial factors may play an important role in ongoing rates of ACs in treated AI (incidence approximately 6-8 ACs/100PY) particularly in young adult females. In order to minimize AC episodes, all barriers to self-management need exploration on an individual patient basis and with regard to the patient population as a whole.

## Introduction

Adrenal insufficiency (AI), including primary AI (PAI), congenital adrenal hyperplasia (CAH) and secondary AI (SAI), is a rare disorder which is associated with a reduced quality of life, increased morbidity and premature mortality (1–6). This condition affects approximately 120/million children and up to 300/million adults in western countries, has a comparable prevalence between the sexes, and increases in prevalence with age (1, 7, 8). In Australia, admission rates for AI and its more severe manifestation, an adrenal crisis (AC), have increased over recent years, especially among females in the young adult age group (8–10). Possible reasons for this include changes in glucocorticoid regimens, increased rates of pituitary adenoma-related AI and difficulties with transition to adult services among patients treated in the paediatric setting (8, 11–13).

AI demands a high level of engagement in self-management by patients. Adults with AI are expected to manage their own daily regimen of glucocorticoid replacement (typically hydrocortisone or cortisone acetate given in two or three divided doses) in addition to maintaining vigilance with regards to signs of a stressor, such as an infection, which requires dose escalation (increased oral glucocorticoids and/or parenteral hydrocortisone) to avert the development of an AC (14, 15). To minimize the risk of an AC, which is characterized by hypotension, hyponatraemia and hyperkalaemia among other features, patients are advised to have extra oral and parenteral glucocorticoid (including injecting equipment) available at all times to enable rapid institution of dose escalation (15). Other recommendations include carriage of a steroid dependency card and wearing medical jewelry denoting an AI diagnosis (15, 16). However, audits of ACs indicate that delays or failures in implementing dose escalation by patients and low levels of use of other preventive measures, such as medical jewelry, are common, despite educational programs aimed at reducing AC incidence (17–20).

Psychological stress is cited as a cause of ACs by some patients (20% in one study) (17, 21), although the characteristics and duration of a perceived stress and the processes through which patient-reported stress may be responsible for an AC have not been thoroughly evaluated (17, 21). It is possible that stress arising from social factors and personal problems such as issues with relationships, finances, and employment, which may be chronic or acute and vary by age and sex, can influence both day-to-day and acute self-management adversely. Due to its pervasiveness, it is also possible that stress may play an important role in the persisting incidence of ACs and the reported delays or failures in dose escalation implementation by educated patients (10, 11, 22).

Given the high burden of AI and AC hospitalizations in the young adult age group, and the recent unexplained very considerable increase in these over the last two decades, especially in women, we aimed to conduct a detailed investigation into patterns of admission among patients with AI in the 20 to 29 years age group. We used admission data on AC and AI hospitalisations to investigate the association between a range of personal and social factors that may influence effective AI self-management and AI-related hospitalisations between the years 2005 and 2021.

## Materials and methods

All admissions to hospitals in New South Wales, Australia, are coded according to the ICD 10 CM-AM (23). Up to 50 diagnoses, considered by the senior clinician to be contributors to the illness episode, are included. Coded data are submitted by hospitals to the NSW Ministry of Health where they are aggregated and stored in the Admitted Patient Data Collection (APDC). For the purposes of this study, deidentified data on all admissions between January 1 2005 and June 30 2021 for adults aged 20-29 years with a principal or comorbid diagnosis of AI were extracted from the APDC for analysis. Eligible diagnostic codes were: E27.1 (primary adrenal insufficiency); E27.2 (adrenal crisis); E23.0 (hypopituitarism); E23.1 (drug induced hypopituitarism); E27.4 (adrenal insufficiency); E25.0 (congenital adrenal hyperplasia); E89.3 (post-hypophysectomy AI). Demographic variables for analysis were age, sex and marital status (classified as married/de-facto or single). Records were excluded if they included codes indicating admissions related to trauma, planned investigations, major surgery (including removal of tumors in the pituitary or adrenal glands) or obstetric/gynaecological indications. Patients with a principal diagnosis of a psychiatric illness were excluded, as it could not be determined whether the admission was because of an acute illness potentially requiring use of dose escalation or directly for psychiatric care. Other exclusions included people visiting Australia; patients with a diagnosis of any malignancy or blood dyscrasias or cystic fibrosis; admissions for rehabilitation; or admissions with a principal diagnosis of an epileptic seizure.

For this analysis, comorbid diagnoses of a psychiatric illness were identified by the following codes: mental and behavioral disorders due to psychoactive substance use (F10-19); schizophrenia, schizotypal, delusional, and other non-mood psychotic disorders (F20-29); mood [affective] disorders (F30-39); anxiety, dissociative, stress-related, somatoform and other nonpsychotic mental disorders (F40-49); behavioral syndromes associated with physiological disturbances and physical factors (F50-59). Other relevant psychosocial factors were identified by the following codes: problems related to employment and unemployment (Z56); problems related to housing and economic circumstances (Z59); problems related to social environment (Z60); other problems related to primary support group, including family circumstances (Z63); problems related to certain psychosocial circumstances (Z64); problems related to other psychosocial circumstances (Z65); problems related to lifestyle (Z72); problems related to life management difficulty (Z73); problems related to care provider dependency (Z74); problems related to medical facilities and other health care (Z75). An additional code for a depressive illness that did not influence the hospital stay (U79.3) was introduced in July 2015 and was included in part of the analysis.

### Data management

As there is no universally agreed definition of an AC, and the ACs identified in this dataset were those diagnosed by the senior managing clinician, an alternate classification, using three common features of an AC: hypotension and/or hyponatraemia and/or hyperkalaemia, which are included as comorbid diagnoses, was also used to identify a severe episode of AI. Records were excluded from this classification if hypotension co-occurred with a diagnosis of comorbid sepsis due to the non-specificity of hypotension in this situation.

For the purposes of analysis, SAI included diagnoses of either hypopituitarism or other/unspecified AI (E27.4). Where relevant diagnoses of psychiatric illness (including alcohol and recreational drug abuse) and other social and personal problems were present in small numbers, data were grouped and presented in aggregate only. As some patients had more than one relevant Z code a ‘Psychosocial score’ (1 point for each relevant code in a record) was calculated to further investigate patterns of psychosocial difficulties and AC/AI admission. In addition, a composite problem score comprising the psychosocial variable (any Z code or no Z code) plus a comorbid psychiatric diagnosis plus a code for drug and alcohol problems (one point for each up to a maximum of three) was also calculated.

### Analysis

Admission rates were calculated using age-sex specific populations extracted from national repositories for each full calendar year of the study, 2005-2020 (https://www.abs.gov.au/statistics/people/population/national-state-and-territory-population/latest-release#data-downloads-data-cubes). Average rates are presented in the mean (sd) format. Three year moving average admission rates were calculated to address the effect of variability and small numbers and are presented in the figures as sequential years from the first averaged estimate. Differences in rates between the sexes or between the first and last years of the study were assessed using the Z test for two proportions. T tests were used to assess differences between continuous variables and Chi square tests were used to assess differences between categorical variables. Pearson correlation coefficients were used to assess relationships between rate changes and rates of stress-related comorbid comorbidities. The analysis was conducted using Excel, SPSS, and GraphPad Prism.

Ethical approval for this study was granted by the HREC of the University of Notre Dame, Australia (Approval #2022-170S).

## Results

There were 877 admissions, 64.9% (n=599) in females, from January 1, 2005 to June 30, 2021 among patients aged 20-29 years. Of these 193 (22.0%) were for an AC; 283 (32.3%) were for PAI; 395 (45.0%) were for SAI; and 43 (4.9%) were for CAH (**Table 1**). There were 226 (25.8%) admissions with one or more of hypotension, hyponatraemia or hyperkalaemia, of which 54 (23.9%) were coded as an AC.

**Table 1:** Characteristics of admissions in 20–29-year-old patients with adrenal insufficiency by sex.

	Male (n/%) N=308	Female (n/%) N=569	Total (n/%) N=877	Significance
Type of Adrenal Insufficiency				
Adrenal Crisis	62 (20.1)	131 (23.0)	193 (22.0)	NS
Primary Adrenal Insufficiency	85 (27.6)	198 (34.8)	283 (32.3)	NS
Congenital Adrenal Hyperplasia	18 (5.8)	25 (4.4)	43 (4.9)	NS
Secondary Adrenal Insufficiency	160 (51.9)	235 (41.3)	395 (45.0)	NS
Signs of an Adrenal Crisis				
Hypotension	43 (14.0)	105 (18.5)	148 (16.9)	NS
Hypernatraemia	41 (13.3)	53 (9.3)	94 (10.7)	NS
Hyperkalaemia	21 (6.8)	16 (2.8)	37 (4.2)	<0.01
Somnolence	3 (1.0)	6 (1.1)	9 (1.0)	NS
Delirium	5 (1.6)	10 (1.8)	15 (1.7)	NS
Abdominal Pain	7 (2.3)	65 (11.4)	72 (8.2)	<0.001
Nausea and Vomiting	24 (7.8)	65 (11.4)	89 (10.1)	NS
**Infection (Any)**	139 (45.1)	226 (39.7)	365 (41.6)	NS
Respiratory	45 (14.6)	47 (8.3)	92 (10.5)	<0.01
Urinary	13 (4.2)	43 (7.6)	56 (6.4)	NS
Cellulitis	8 (2.6)	13 (2.3)	21 (2.4)	NS
Gastroenteritis	53 (17.2)	103 (18.1)	156 (17.8)	NS
Diabetes Mellitus (DM)*	45 (14.6)	126 (22.1)	171 (19.5)	<0.01
Type 1 DM	26 (8.4)	81 (14.2)	107 (12.2)	<0.05
Type 2 DM	17 (5.5)	40 (7.0)	57 (6.5)	NS
**Diabetes Insipidus**	15 (4.9)	26 (4.6)	41 (4.7)	NS
Psychiatric Comorbidity				
Anxiety	13 (4.2)	18 (3.2)	31 (3.5)	NS
Depression	7 (2.3)	15 (2.6)	22 (2.5)	NS
Alcohol and other drug use	–	35 (6.2)	40 (4.6)	<0.01
Anxiety/Depression/Psychosis	19 (6.2)	33 (5.8)	52 (5.9)	NS
**Social and Personal Circumstances (1 or more)**	46 (14.9)	148 (26.0)	194 (22.1)	<0.001
Lifestyle Problems	24 (7.8)	111 (19.5)	135 (15.4)	<0.001
Social Problems	15 (4.9)	27 (4.7)	42 (4.8)	NS
Personal Factors (Z91)	–	–	9 (1.0%)	NS
Family Problems (Z63)	–	–	10 (1.1%)	NS
**Depression (U79.3)#**	9 (2.9)	74 (13.0)	83 (9.5)	<0.001
**Marital Status**	33 (10.7)	109 (19.2)	142 (16.2)	<0.01

*Includes 7 Other DM (E13).

# from July 2015 onwards.

NS, non significant.

The average admission rate of 63.5(38.0)/million/year for females was almost twice the average male rate of 34.0 (13.4)/million/year (p<0.01). Females comprised the majority (67.9%, n=131) of AC admissions, corresponding to an average female AC admission rate of 14.7 (12.3)/million/year, while the average male rate was 6.75 (3.77)/million/year (p=NS).

**Figure 1:**
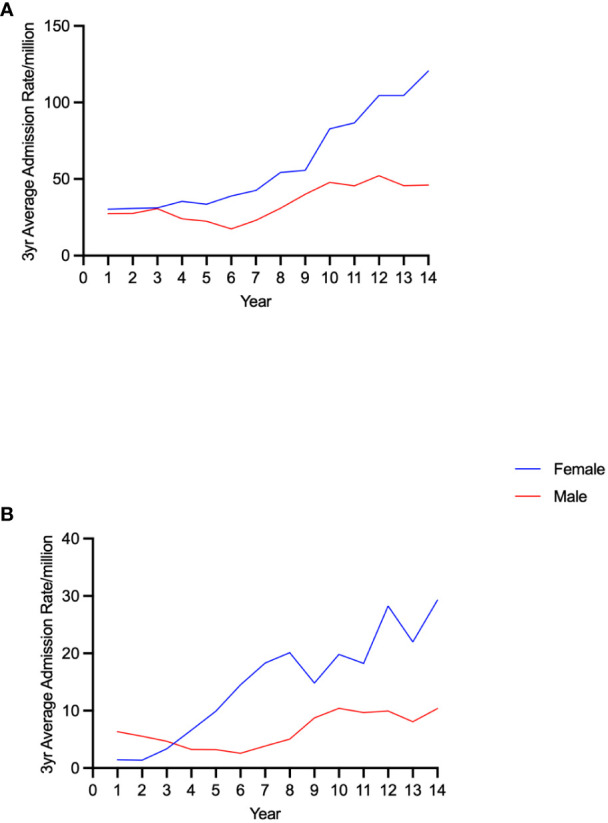
Trends in admission rates for adrenal insufficiency **(A)** and adrenal crisis **(B)** by sex in 20-29 year old individuals.

**Figure 2:**
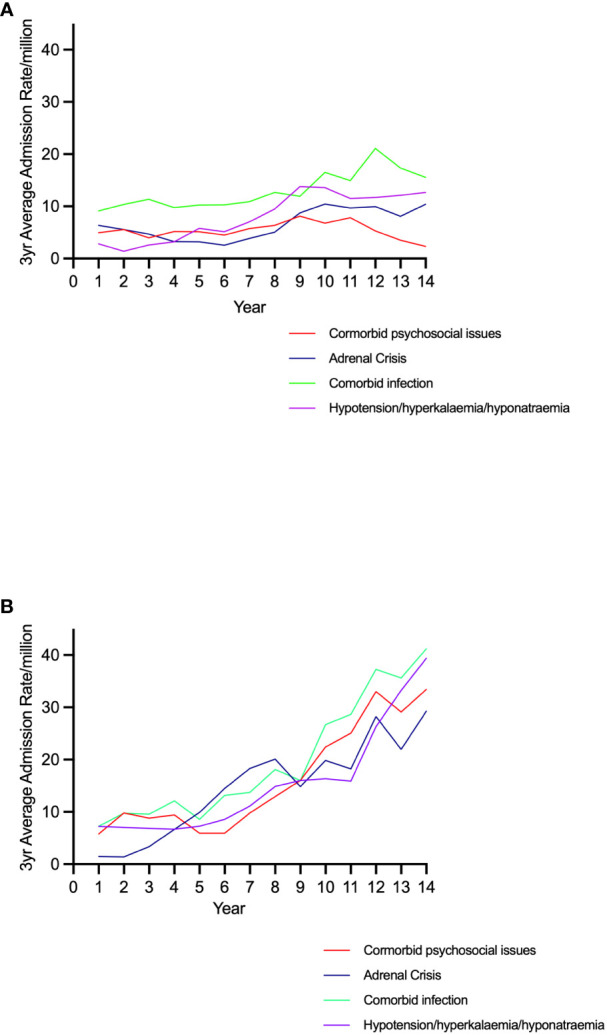
Trends in admission rates for adrenal crisis, signs of acute adrenal insufficiency, comorbid infection, and comorbid psychosocial issues by sex in 20-29 year old individuals. **(A)** Males **(B)** Females.

Admission rates for AI increased over the study period in both sexes (**Figures 1A, B**). The three-year moving average rate among females increased by 298.0%, from 30.3 to 120.6/million. This was higher than that in males, which increased by 68.2%, from 27.4 to 46.1/million (both p<0.001) (**Figures 1A, B**). There was a greater increase, of 1992.9%, in AC-related admissions in females (from 1.4 to 29.3/million, p<0.001) which was larger than that in males, 65.1% (from 6.3 to 10.4/million, p<0.01) (**Figures 1A, B**). Admissions with one or more of hypotension, hyponatraemia or hyperkalaemia also increased in women (by 488.9%, from 7.2 to 42.4/million, p<0.001) and to a lesser extent in males (by 259.2%, from 3.5 to 12.7/million, p<0.001) (**Figures 2A, B**). Only 31 (22.4%) of all AC admissions had the underlying cause of AI included in the record.

### Comorbid conditions

Overall, 41.6% (n=365) of the admissions were associated with an infection and infectious gastroenteritis was recorded in 17.8% (n=156) (**Table 1**). Respiratory tract infection (n=92, 10.5%), urinary tract infection (n=56, 6.4%), and sepsis (n=42, 4.8%) were less common.

Diabetes mellitus (DM) was a common comorbid endocrine diagnosis, with T1DM being recorded in 12.2% (n=107) of the admissions [38.3% (n=41) of these had diabetic ketoacidosis and 17.8% (n=19) had hypoglycaemia]. Fewer patients had T2DM (6.5%, n=57) and comorbid diabetes insipidus (DI) was recorded in 41 (4.7%) admissions. Comorbid major psychiatric disorders (anxiety, depression, or schizophrenia) were found in 52 (5.9%) of the admissions, while alcohol and/or other drug use was identified in 40 (4.6%) admissions (**Table 1**). Three patients were coded as having “poisoning by, adverse effect of and underdosing of glucocorticoids and synthetic analogues” (T38.0).

Comorbid T1DM was more frequent among female patients, affecting 14.2% (n=81) of the female admissions compared with 8.4% (n=26) of male admissions (p<0.05). There was a significant association between T1DM and an AC in females only (19.8%, n=26 with an AC vs 12.6%, n=55 without, p<0.05) and, while there were more females with T1DM in the group with signs of an AC (18.7%, n=28 vs 12.6%, n=53) this difference was not significant. By comparison, neither an infection nor gastroenteritis were associated with admission for an AC or the alternate severe AI classification in either gender. Similarly, a comorbid diagnosis of psychiatric illness was not significantly associated with an AC or with classification in the severe symptom category. In contrast, very few patients (7.3%, n=3) admitted with comorbid DI were recorded as having an AC although a greater proportion (29.3%, n=12) were in the alternate signs of severe AI category.

### Other social and personal factors

A comorbid diagnosis of at least one personal or social problem was found in 194 (22.1%) admissions. Of these, a problem associated with lifestyle factors (Z72) was the most common (15.4%, n=135), followed by social problems (Z60) in 4.8% (n=42) of admissions (**Table 1**). Nine (1.0%) admissions were associated with ‘personal factors’; non-compliance with therapy was rare (n=5, 0.6%); problems with care dependency were recorded in 8 (0.9%); problems with housing in 8 (0.9%); and 10 (1.1%) admissions were associated with family problems.

**Figure 3:**
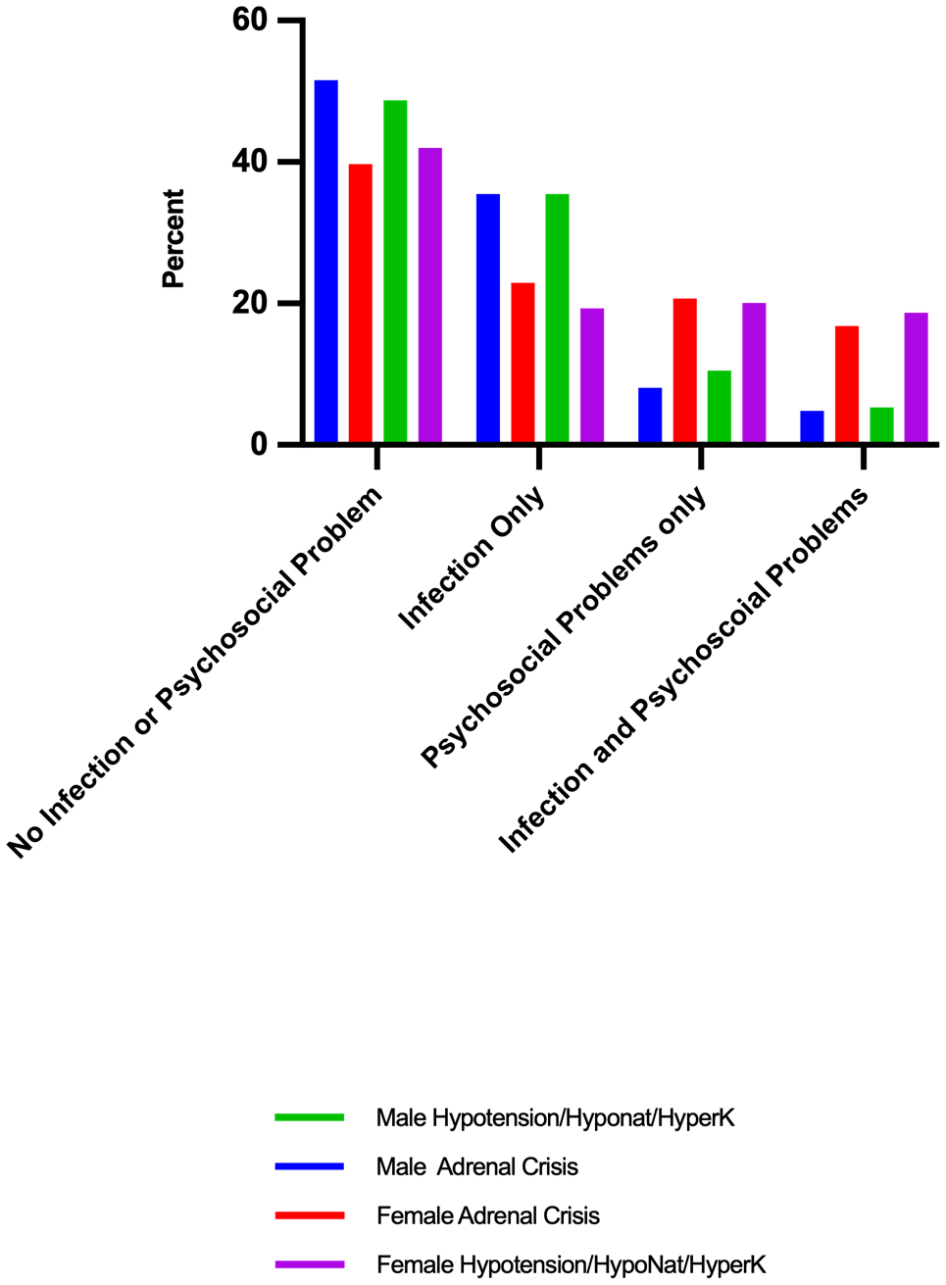
Proportion of patients admitted for severe symptoms of adrenal insufficiency with an infection or psychosocial problems: by sex and adrenal crisis diagnosis or signs of severe adrenal insufficiency.

Females had a greater proportion of admissions (26.0%, n=148) in which at least one z code was recorded as affecting the episode of care than males (14.9%, n=46, p<0.001) and had fewer admissions without any comorbid psychosocial problems or an infection (**Figure 3**). Having one or more personal/social problems was significantly associated both with an AC (37.4%, n=49 vs 22.6%, n=99, p<0.001) and with the signs of an AC category (37.3%, n=56 vs 22.0%, n=92, p<0.001) in females but there was no such association in males (**Figure 3**). Females also had a higher mean psychosocial score than males [0.32 (0.56) and 0.18 (0.43), respectively, p<0.001] and females with an AC had a higher mean psychosocial score [0.46 (0.67)] than those without an AC [0.27 (0.51), p <0.01]. Similarly, female patients with cardinal signs of acute AI had a higher mean psychosocial score [0.44 (0.61)] relative to those not in this category [0.27 (0.53), p<0.01]

Patients with comorbid DI had few comorbid psychosocial issues (7.3%, n=3) while those with T1DM had a greater proportion (24.3%, n=26). Patients in a married/de-facto relationship comprised 16.2% (n=142) of the study sample. Fewer female patients with an AC were in a married/de-facto relationship (10.7%, n=14) than those without an AC (21.7%, n=95, p<0.01) but there was no such relationship among male patients.

Females who had an admission for an AC also had a significantly higher composite psychosocial, psychiatric and drug/alcohol score [0.47 (0.67)] than those without an AC [0.32 (0.62) p<0.05]. Similarly, females in the category of signs of an AC had higher mean composite problem score [0.51 (0.71)] than those without [0.30 (0.29) p<0.01]. No comparable associations were found in male patients.

Admissions associated with at least one psychosocial problem increased over the study period, from 5.8 to 33.4/million (p<0.001) in females but remained unchanged in males (4.9 to 2.3/million, p=NS) (**Figures 2A, B**). Changes in psychosocial score rates were correlated with AI (r=0.98, p<0.001) and AC admission rates (r=0.82, p< 0.001) in females but not males (AI: r=0.10 and AC r=0.0.15, p=NS). Similarly, changes in admission rates for any of hypotension/hyponatraemia/hyperkalaemia were correlated with psychosocial score rates (r=0.91, p<0.001) in females but not among males (r=0.25, p=NS)

Eighty-three (9.5%) admissions from 2016 onwards were associated with a depressive illness (U79.3) that was not considered to have contributed directly to that episode of illness. This was more common in females (13.0%, n=74) than males (2.9%, n=9, p<0.001).

## Discussion

This population-based study of hospitalizations for an acute medical illness among young adults aged 20 to 29 years, demonstrated that rates of admission with an AI or AC diagnosis have increased substantially, especially among females. AC rates increased to a greater extent than AI related admissions in females but not in males, reflecting higher rates of diagnoses of severe illness in females. Infection was associated with hospitalization in 41.6% of all admissions and in 39.9% of AC-related hospitalizations. By comparison, admissions in which the social and personal circumstances of the patient were considered to have contributed to the illness episode comprised 22.1% of all admissions and 29.0% of AC admissions. These featured more prominently in females among whom 37.4% of AC admissions were associated with personal stressors, and in whom an increase in the prevalence of personal stressors was strongly correlated with higher AC and AI admission rates. Comorbid psychiatric illness and drug and alcohol use, which were considered contributors to the hospitalization, were found in fewer than 5% of admissions each, while a depressive illness, which was present but not considered directly contributory to the admission, was more common especially among women.

The considerable increase in rates of hospitalization among females in this study is consistent with results of an earlier nationwide analysis that examined admission rates for a principal diagnosis of an AI or AC in this age group (8, 10). While the reasons for this have not been established, changes to the underlying incidence of AI or increases in the prevalence of factors which lead to the need for hospital treatment among AI patients, or a combination of these, are likely to be responsible. PAI and pituitary adenomas are more common diagnoses in females than males at this life stage and increased adenoma detection rates, following investigations for unrelated issues or for fertility or menstrual problems, may have resulted in an increased incidence of SAI in females (24, 25). However, the substantial increase in AC admissions suggests that other factors are also influencing these trends. Changes to glucocorticoid replacement therapy utilising shorter acting glucocorticoids (hydrocortisone and cortisone acetate) prescribed at lower daily doses have been associated epidemiologically and in cohort studies with increased AC rates (8, 21). In addition, problems managing transition from paediatric to adult health services, which have been shown to lead to higher rates of complications in patients with other chronic diseases such as T1DM, may also contribute to a rise in AC incidence in this age group (12, 26–28). However, the results of the present study suggest that social and personal factors also contribute to AC events, mediated through problems either with adherence to maintenance regimens or with implementing dose escalation or a combination of these.

The findings of this study demonstrate the importance of considering the management of young adult patients with AI in a wider context. The ability to self-manage effectively is necessarily located within the social and personal setting of each patient. Susceptibility to the effects of stressors on young adult females, suggested in this analysis, indicates that social and personal vulnerabilities may be more influential in some subgroups of patients than others. Factors removed from the patients themselves, such as fluctuations in the economic environment impacting on aspects of daily life, encompassing employment, costs of living, including of medical consultations and pharmaceuticals, and housing affordability may act as stressors and impact on the ability of patients to self-manage their AI. Social dislocation or variable accommodation arrangements, typically occurring when young adults move out of home, may also have a role in the illness experience of this age group. Education of family and household members regarding dose escalation and administration of emergency hydrocortisone assists in management of patients with AI, especially in the paediatric age group, and may not be consistently available to patients in the emerging adult years (29). A positive effect of such support is suggested in this analysis by the higher proportion of married/de-facto female patients in the group who did not experience an AC.

Diabetes mellitus was a common comorbidity in the patients in this study and comorbid T1DM was associated with ACs in females but not males. Although evidence on the effect of comorbid T1DM on AC occurrence is variable, it is possible that the demands on patients imposed by the requirement to self-manage simultaneously these two conditions during stress may increase the risk of acute illness (19, 30). Comorbid DI has been associated with increased admissions for acute AI-related illness in children but this was not found to be the case in the young adults in this study (31). While the reasons for this are not known, it is possible that patients with DI have more complex care needs and retain greater involvement with family and other carers as they emerge from adolescence. This may be reflected in the low prevalence of z codes in the records of these admissions.

There is no universally accepted AC definition and the definition used in this dataset refers to that used by individual clinicians managing each patient (15). For this reason, an alternate classification based on three key features of an AC (hypotension, hyponatraemia and hyperkalaemia) was used in this study (15). More patients had at least one or more of the key signs of an AC than were diagnosed as having had an AC and only approximately 25% of diagnoses overlapped, suggesting that there is variability in definitions. Despite this, in this analysis, population-based rates of admission for severe AI-related illness increased, irrespective of the definition used. A limitation of this study is that the data were not matched at the individual patient level and, therefore, repeat admissions for individual patients could not be identified. Evidence indicates that some patients have more than one AC admission but that it would be likely that only a relatively small number of patients had two or more admissions during the time period of the study and that this would not have affected the overall conclusions (17–19).

By placing self-management of AI into the wider context of social and personal factors which influence the health and well-being of patients, this study suggests that there are inevitable limitations of an approach to patient care in which education is provided without additional support for patients with vulnerabilities. The results of this study provide an indication of some of the complexities of modern AI management by placing the need for hospital treatment in wider sociocultural setting. New evidence from this investigation suggests that psychosocial factors may play an important role in ongoing rates of ACs in treated AI (incidence approximately 6-8 ACs/100PY) particularly in young adult females. In order to minimize AC episodes, all barriers to self-management need exploration on an individual patient basis and with regard to the patient population as a whole.

## Data availability statement

The datasets presented in this article are not readily available because they include information on individual patients and were released by the data custodians only to the named recipient for the purpose of this research. Requests to access the datasets should be directed to rosemary.louise.rushworth@nd.edu.au.

## Ethics statement

The studies involving humans were approved by HREC University of Notre Dame, Australia. The studies were conducted in accordance with the local legislation and institutional requirements. The ethics committee/institutional review board waived the requirement of written informed consent for participation from the participants or the participants’ legal guardians/next of kin because data were provided from a large database that was de-identified and no individuals could be identified.

## Author contributions

All authors listed have made a substantial, direct, and intellectual contribution to the work and approved it for publication.
